# 3D images as a field grader training tool for trachomatous trichiasis: A diagnostic accuracy study in Ethiopia

**DOI:** 10.1371/journal.pntd.0007104

**Published:** 2019-01-24

**Authors:** Jeremy J. Hoffman, Esmael Habtamu, Hillary Rono, Zerihun Tadesse, Tariku Wondie, Temesgen Minas, Bizuayehu Gashaw, E. Kelly Callahan, David MacLeod, Matthew J. Burton

**Affiliations:** 1 International Centre for Eye Health, London School of Hygiene and Tropical Medicine, London, United Kingdom; 2 NIHR Biomedical Research Centre for Ophthalmology, Moorfields Eye Hospital and UCL Institute of Ophthalmology, London, United Kingdom; 3 Ophthalmology Department, Kitale District Hospital, Kitale, Kenya; 4 The Carter Center, Addis Ababa, Ethiopia; 5 Amhara Regional Health Bureau, Bahir-Dar, Ethiopia; 6 The Carter Center, Atlanta, Georgia, United States of America; 7 Tropical Epidemiology Group, London School of Hygiene and Tropical Medicine, London, United Kingdom; RTI International, UNITED REPUBLIC OF TANZANIA

## Abstract

**Background:**

Trachomatous trichiasis (TT) will continue to develop among those people who have had repeated infections after active trachoma is controlled. Detecting and treating affected individuals will remain necessary for years; a long “tail” of incident cases is anticipated. As the prevalence of TT declines, there will be fewer cases available for training trachoma graders (TG), necessitating alternative methods.

**Methodology/Principal findings:**

Prospective, diagnostic accuracy study assessing sensitivity and specificity of 3D and 2D photography as a tool for training TG to detect TT. Individuals with TT in Ethiopia were examined, and 2D and 3D clinical images taken. Images were independently graded by four graders for presence or absence of trichiasis and compared to field grading. We recruited 153 participants. Clinical assessments and images were available for 306 eyes. Trichiasis was identified in 204 eyes by field grading. Image grading was performed on a selection of 262 eyes (131 with trichiasis). Most eyes with trichiasis had minor trichiasis (94/131). Pooled sensitivity was 88.3% (3D) and 98.0% (2D); pooled specificity was 59.8% (3D) and 26.8% (2D). 3D photo grading was 33.0% more specific than the 2D photo grading (p = 0.0002). The overall Kappa scores were 0.48 (3D) and 0.25 (2D). We trained 26 novice TG in Ethiopia using 3D images. They were tested on a 3D images set and had 71.4% agreement (kappa 0.46), relative to an expert. They were then tested examining 50 people, and had 86.8% agreement (kappa 0.75). We also tested 27 experienced TG on the same cases (86.4% agreement, kappa 0.75). There was no difference in performance between groups (p = 0.76). All participants preferred 3D over 2D images for training.

**Conclusions/Significance:**

The slightly higher sensitivity of 2D photos comes at considerable cost in specificity. Training with 3D images enabled novice TG to identify cases as well as experienced TG. 3D were preferred to conventional 2D photos for training. Standardized 3D images of TT could be a useful tool for training TG, in settings where there are now few TT cases.

## Author summary

Trachomatous trichiasis (TT) is the in-turning of eyelashes, which leads to sight loss. Control programmes are training health workers to recognise and refer TT for surgery. Currently, training uses a mix of 2D images and examination of live patients. With the decline in TT prevalence, there will be too few TT patients available for this training. Here we explored the possibility of using 3D images of eyes with TT to train and test new trachoma graders. We found that experienced graders were more accurate using 3D than 2D photos, compared to “live grading”. We found that novice trachoma graders could be successfully trained using 3D images, without seeing live patients; and were as reliable as experienced graders when tested in “live grading” on a mixture of people with and without TT. Therefore, we think that 3D photos offer an alternative training tool for programmes, particularly when there are too few people with TT to engage in training activities.

## Introduction

Trachoma remains the commonest infectious cause of blindness worldwide.[1] The World Health Organization (WHO) Alliance for the Global Elimination of Trachoma (GET2020) aims to eliminate the disease as a public health problem by the year 2020.[2] The two key clinical parameters used to guide programme decisions and the assessment of elimination are the prevalence of trachomatous inflammation—follicular (TF) conjunctivitis in children and trachomatous trichiasis (TT) in adults. These clinical signs form part of the WHO Simplified Trachoma Grading System, which was designed for field grading by non-specialists, and is widely used in surveys to measure the disease prevalence.[3]

The Global Trachoma Mapping Project (GTMP), which mapped nearly all accessible suspected trachoma endemic districts, developed a protocol to train trachoma graders to reliably recognize these signs.[4,5] This built on earlier training protocols used by trachoma programmes in Ethiopia, Nigeria and South Sudan.[6–8] As countries approach the elimination targets and need to demonstrate sustained achievement, a similar methodology is being applied, through Tropical Data, with additional surveys following the cessation of intervention programmes. Tropical Data is a WHO led survey methodology and data management platform developed partly out GTMP, after GTMP was completed in 2015. The GTMP/Tropical Data trachoma prevalence survey training for graders is conducted over five days, the first two of which are an intensive classroom- and field-based “grader qualifying workshop”. [9] In order to qualify as a grader, candidates need to pass (kappa ≥0.7) a photograph-based inter-grader agreement (IGA) test comprising 50 images showing the presence or absence of TF. Subsequently, they need to pass a field IGA test (kappa ≥0.7 against a grader trainer) on 50 children of whom at least 5 have TF. Tropical Data has also developed a TT-only survey training manual, which includes an Objective Structured Clinical Examination (OSCE) using photographic images (2D or 3D) to assess trainees’ skills in TT grading, as the number and availability of people with trichiasis is too low to assess via an IGA assessment. [10]

In 1990 West and Taylor proposed that using still images was a valid and reliable tool for grading trachoma.[11] This was used to assess the appearance of the tarsal conjunctiva, but not TT. Since then, there have been a number of studies comparing field grading to photographic assessment.[12–14] The Kappa-values in these studies ranged from 0.44 to 0.75. However, none of these studies have compared the field and photograph grading of TT. A recent trial comparing two alternative trichiasis surgery procedures used two dimensional (2D) clinical images to assess the presence of TT following surgery.[15] In this study the photographic grading result was highly concordant with the field grading. However, TT was slightly “over-called” from these images compared to the field-grading; this was thought to be due to the two-dimensional nature of the images, which can give the impression that lashes overlying the globe are touching when there is actually a small gap.

Three-dimensional (3D) images may be able to reduce this limitation of 2D images by providing an additional perspective on whether the eyelashes close to the eye are actually touching the globe. There have been no previous reports of the use of 3D photography to assess trichiasis. In this study we investigated whether this might be a useful tool in the training and assessment of graders within a trachoma control programme, especially during the anticipated long “tail” of incident TT, following the control of active disease. We initially compared the masked grading of 2D and 3D images to the “live” field grading of the same eyes, for the presence of TT with lashes touching the eye. We then trained a cohort of trichiasis graders using 3D photography and compared their performance with experienced trichiasis graders.

Our objectives were to firstly investigate the relative diagnostic accuracy of 2D and 3D images, compared to “live” grading. Secondly, to evaluate the utility of using 3D images within training programmes for teaching the novice trainees how to detect TT and compare their performance in “live” grading by experienced graders.

## Materials and methods

### Ethics statement

This study was approved by the Ethiopian National Health Research Ethics Review Committee, the London School of Hygiene & Tropical Medicine Ethics Committee and Emory University Institutional Review Board. It conformed to the tenets of the Declaration of Helsinki. Written informed consent in Amharic was obtained before enrolment of participants. If a participant was unable to read and write, the information sheet and consent form were read to them and their consent recorded by thumbprint in the presence of an independent witness. The study took place in Amhara Region, Ethiopia. It was conducted in two parts: (1) comparison of field to photographic grading; (2) evaluation of 3D images within a training programme. Only adults were recruited into the study.

### Comparison of 2D and 3D masked photography grading to field grading

In this prospective study, consecutive adults with upper lid TT in one or both eyes were recruited through community-based screening conducted at community health centres in three districts of West Gojam Zone. TT was defined as one or more lashes touching the globe. Both eyes were examined by a single experienced field grader (EH) using 2.5x binocular loupes with a torch and graded using the Detailed WHO FPC Grading System.[16] Eyelids were graded for the presence or absence of TT and the number of lashes touching were counted. Standardised 2D and 3D images were taken using a Nikon D90 digital SLR camera with Loreo 3D macro lens and Nikon SB-R200 flash units. Images were taken in primary gaze and up-gaze. We found substantial dermatoblepharochalasis was common and frequently obscured the view of the lid margin and lashes. Therefore, the skin of the upper eyelid was supported with a swab shaft to prevent it resting heavily on the lashes. Care was taken to ensure that this did not cause external rotation of the lid margin that might affect whether or not lashes touched the globe.

**Fig 1:**
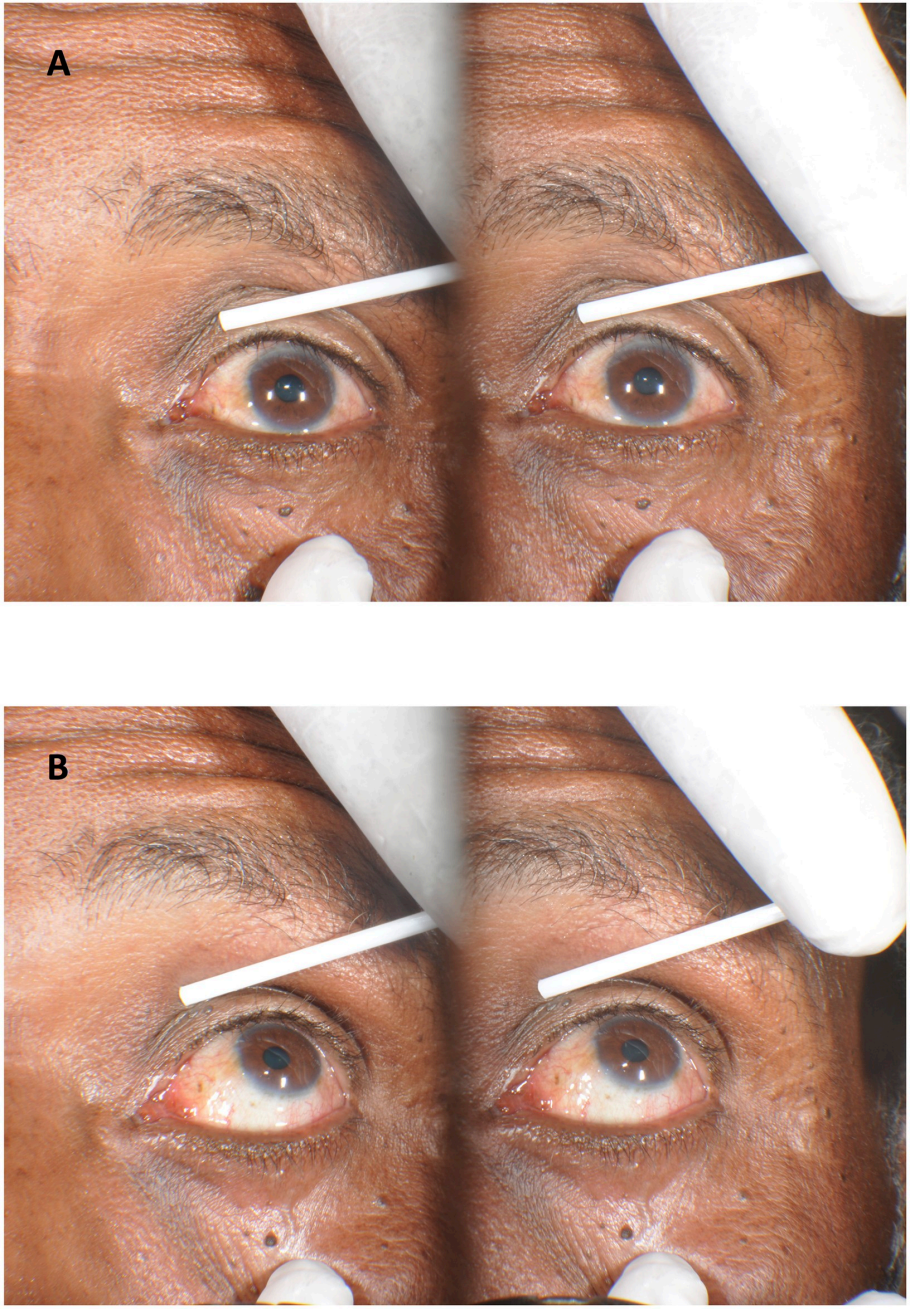
Images of an eye with TT taken using a Loreo 3D macro lens; (A) primary position of gaze, (B) up gaze.

The Loreo 3D system works by transposing two images within the beam-splitter into a parallel format output. This results in a pair of “split images” (Fig 1). This image pair can then be viewed using a low-cost parallel format 3D-image viewer which incorporates two prismatic lenses, allowing someone with stereoscopic vision to fuse the images and view the image in 3D. The resulting image size on the retina is similar to that obtained when viewing a patient with 2.5x loupes.

Comparisons were made between the field grading (EH) for the same eye and the image grading by four independent graders with experience in trachoma field grading. For the image set that was used in this comparison, poor quality images (out of focus, movement artefact, over/under exposed) were excluded. In addition, we randomly excluded images of eyes with TT until we were left with an approximately equal number of images with and without TT. The orders of the both 3D and 2D image sets were randomised and all images were relabelled. The 3D images were viewed on a MacBook Pro 15” with Retina Display computer (Apple) using Loreo 3D Pixi-Viewer glasses under standardised conditions: monitor set to full brightness in a darkened room and no changes made to the display settings. For the 2D assessment, the left-hand image of the two “split images” was used without the 3D glasses. Grading of all the 2D and 3D images was done separately. Each eye was evaluated using the primary gaze and up-gaze images. Graders were asked to specify whether they thought there were any lashes touching the globe. For the counting of lashes, the highest number of lashes touching the eye seen in either primary or up-gaze was recorded as the result.

### Evaluation of 3D images within a training programme

In February 2018, 26 health professionals (17 Health Officers, and 9 BSc Clinical Nurses), hereafter referred to as Trichiasis Graders (TG), with no prior training or experience of TT case identification were recruited from 17 districts of West Gojam Zone, Amhara Region, and were enrolled on a four-day training programme for TT case identification using 3D images. The mean age of the TGs was 26 years (Range 21–40 years), 18 were male and 8 were female.

The training was based on the Amhara Region Integrated Eye Care Worker (IECW) training manual and incorporated all the components of IECW training except for trichiasis case identification training using live subjects. This training included prevention of blindness and primary eye care, anatomy of the eye, common eye conditions, trachoma, TT case mobilisation, eyelid examination techniques using magnifying loupes. Trainees were initially shown clinical images of trachoma in 2D, including TT, projected onto a large screen. Then they were trained in the identification of trichiasis using a series of 3D pictures.

**Fig 2:**
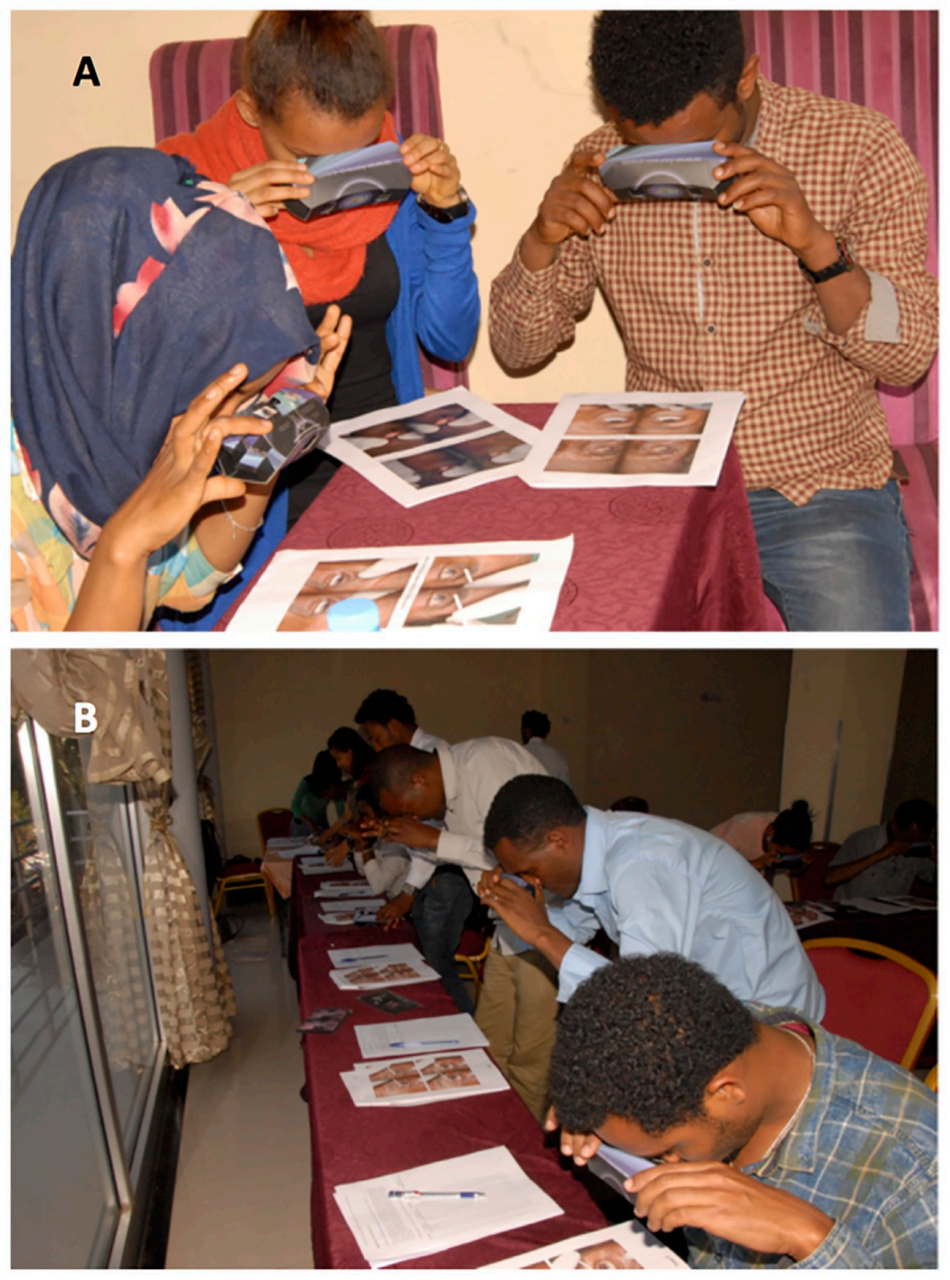
Trichiasis graders (a) practicing grading trichiasis using 3D images, and (b) performing the intergrader 3D image test.

The trainees were shown how to view 3D images printed on paper using the Loreo Pixi-Viewer 3D glasses (Fig 2a). Once they were confident with obtaining a 3D view, they were shown a series of 3D images of eyes with and without trichiasis over one day of intensive training. They were taught how to grade whether trichiasis was present or not and to count the number of eyelashes touching the eye if trichiasis was present. On the final training day, all trainees were tested in an intergrader assessment (IGA) using a set of 3D colour printed images of 50 eyes with and without trichiasis (Fig 2b). This set of images had been selected from those used in Part 1. We only included images for which all four experienced graders agreed with the field grading on the presence or absence of trichiasis. The image set contained 28 eyes without trichiasis and 22 cases with trichiasis. For each eye two 3D pictures were printed on one page showing primary position and up-gaze (Fig 1). The trainees were asked to grade the eye for the presence or absence of trichiasis, and then to count the number of lashes touching the eye if TT was thought to be present. They were allowed 30 seconds per image (1 minute per eye), equating to a test of 50 minutes in duration. Images with only evidence of epilation, but without lashes currently touching, were graded as having “no trichiasis”.

Immediately after the IGA the trichiasis graders were asked to complete an evaluation form on the 3D training. The evaluation form included questions on the ease of using 3D glasses, subjective comparison of 2D and 3D images, future application of 3D image-based trichiasis graders training, and suggestions for improvements.

After the completion of the 3D training and IGA, the trichiasis graders were then taken to the field to assess 50 patients (one eye per patient) with and without trichiasis, using 2.5x magnifying loupes and a torch. In this “live” clinical assessment test they were allowed 90 seconds per patient. There were 23 people (eyes) with trichiasis and 27 people (eyes) without trichiasis. The trichiasis graders were asked to record presence or absence of trichiasis, and to count the number of lashes touching the eye, if trichiasis is present. Their results were compared to the grading given by an expert trachoma grader (EH) on the same day.

Immediately after the trainee test, a separate group of 27 experienced IECWs (8 Health Officers, and 19 BSc Clinical Nurses), with a mean age of 28 years (range 24–36 years), from 17 districts of Wet Gojam Zone, Amhara Region, examined and graded the same group of 50 patients using the same procedure. This was done to compare the grading quality of trichiasis graders trained using 3D images to the grading quality of experienced IECWs, most of whom had previously been involved in trachoma impact assessment surveys. At the end of the exercise, all subjects were re-graded by the expert trachoma grader.

### Analysis

Data were double-entered into an Access database (Microsoft) and transferred to Stata 14 (StataCorp) for analysis. For the first part of the study we calculated for each grader the sensitivities, specificities, positive predictive values (PPV), negative predictive values (NPV), overall percentage agreement and Kappa scores relative to the field grading (EH). Estimated values for sensitivity and specificity were obtained using logistic regression with a random effect included for the rater, the mean kappa score was estimated by taking the mean of the Fisher Z-transformed kappa scores, then back-transforming. P-values comparing sensitivities and specificities for 2D versus 3D images were calculated using the Z-test. For the second part, we compared the results of the novice TG image grading and live clinical assessments to the grading of the same patients by the expert grader (EH). Similarly, we compared the results of the experienced ICEWs to those of the expert grader. We plotted Hierarchical Summary Receiver Operating Characteristic (HSROC) curves for the relationship between the reference field grading (EH) and the trainees’ grading of the 3D images.

## Results

### Comparison of 2D and 3D masked photography grading to field grading

**Fig 3:**
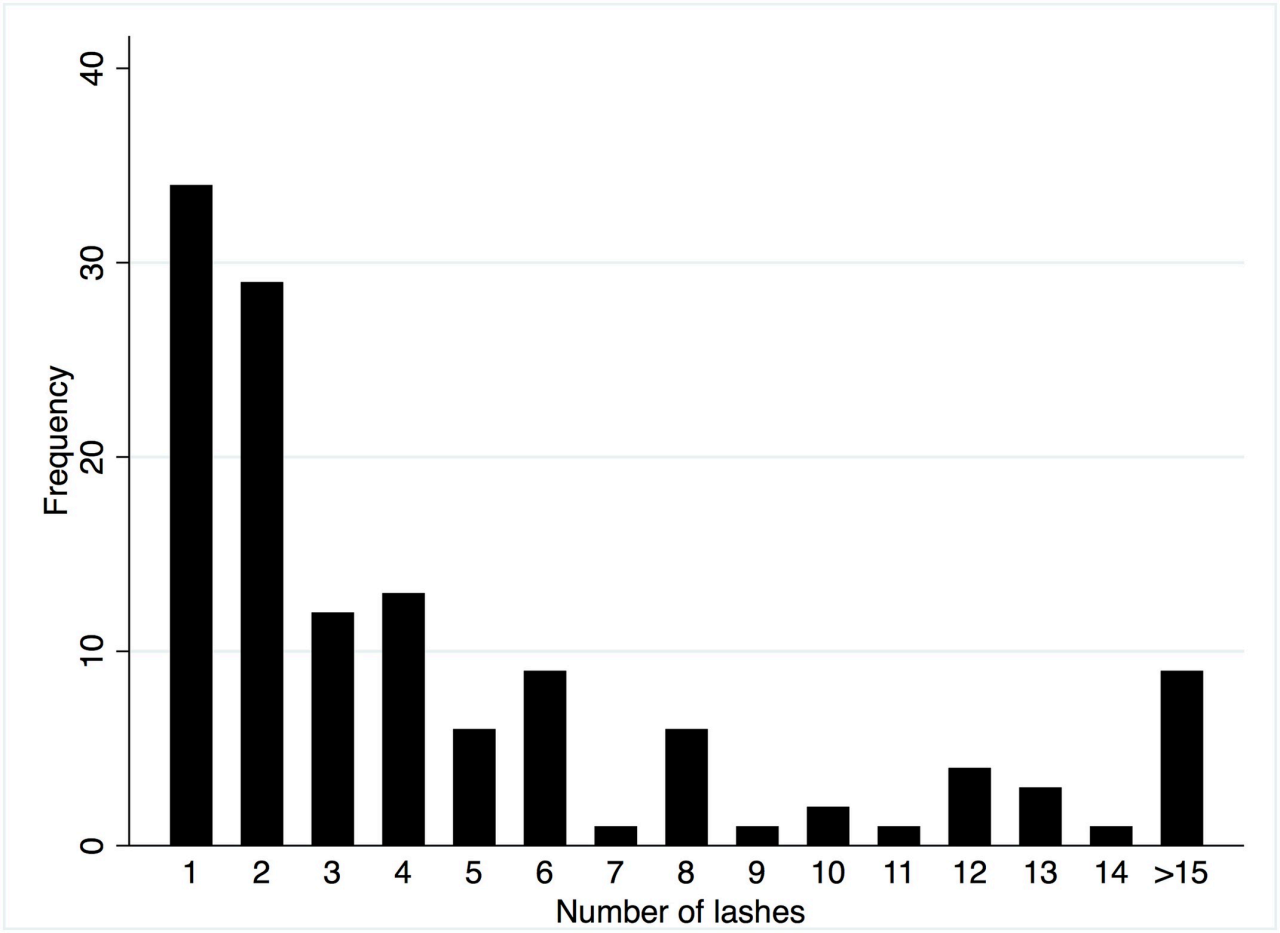
Distribution of the number of lashes touching the globe for eyes identified as having current trichiasis by field grading.

We recruited, examined and photographed the eye of 153 people for this study. Their mean age was 50.9 years SD 14.0, range 18–80) and 96 (62.8%) were female. We selected 260 good quality eye images for masked grading, of which 131/260 (50.4%) eyes had trichiasis and 129/260 (49.6%) did not have lashes touching the globe at the time of examination. Among eyes with current TT, the mean number of lashes touching the globe was 5.16 (SD 6.28, median 3, range 1–40). The distribution of the total number of lashes touching the eyes with trichiasis is shown in Fig 3.

The sensitivity and specificity, PPV and NPV for the 3D image grading compared to the field grading are presented in Table 1. The pooled estimates of sensitivity and specificity were 88.3% (95% CI 84.4–91.4%) and 59.8% (95% CI 46.2–72.1%), respectively. The corresponding results for the 2D photos are also presented in Table 1. Their pooled sensitivity and specificity were 98.0% (95% CI 91.4–99.6%) and 26.8% (95% CI 17.2–39.2%), respectively. There was a statistically significant difference in both the sensitivity and specificity between 2D and 3D images for each grader (Table 1). Overall, the sensitivity was slightly higher (9.7%, p = 0.0004) for the 2D images and specificity substantially higher for 3D images (33.0%, p = 0.0002). This suggests that although 2D grading was slightly more sensitive, this was at the expense of reduced specificity. There was also slightly better overall agreement between the grading of 3D images (73.9%) and the field grading, compared to the 2D image (62.8%) grading. One grader (Grader 3) tended to over-grade TT, resulting in a lower specificity for both 2D and 3D photos.

**Table 1:** Comparison of 3D and 2D photo grading to field grading: Sensitivity, specificity, negative predictive value, positive predictive value and agreement. Difference in the sensitivity and specificity between 2D and 3D photos, relative to field grading (EH).

Test Characteristic	Grader				Pooled Results
	1	2	3	4	
**3D Photos**					
Sensitivity (%)	90.1	89.3	90.8	82.4	88.3 (CI 84.4–91.4)
Specificity (%)	56.6	72.1	38.8	69.8	59.8 (CI 46.2–72.1)
PPV (%)	67.8	76.5	60.1	73.5	69.5
NPV (%)	84.9	86.9	80.6	79.6	83.0
Agreement (%)	73.5	80.8	65	76.2	73.9
Kappa	0.47	0.61	0.29	0.52	0.48
95%CI	0.37–0.57	0.52–0.70	0.19–0.40	0.42–0.63	0.25–0.66
p-value	<0.0001	<0.0001	<0.0001	<0.0001	0.009
**2D Photos**					
Sensitivity (%)	96.9	96.9	100	93.3	98.0 (CI 91.4–99.6)
Specificity (%)	34.1	35.7	11.6	31	26.7 (CI 17.2–39.2)
PPV (%)	59.9	60.5	53.5	58	58.0
NPV (%)	91.7	92	100	83.3	91.8
Agreement (%)	65.8	66.5	56.1	62.7	62.8
Kappa	0.31	0.33	0.12	0.25	0.25
95%CI	0.22–0.40	0.24–0.42	0.06–0.17	0.16–0.34	0.10–0.40
p-value	<0.0001	<0.0001	<0.0001	<0.0001	0.014
**Difference between 2D and 3D**					
Sensitivity difference	6.8%	7.6%	9.2%	11.5%	9.7%
95%CI	1.0–12.8	1.6–13.7	4.2–14.1	3.8–19.1	5.1–14.3
p-value	0.024	0.015	0.0004	0.004	0.0004
Specificity difference	-22.5%	-36.4%	-38.7%	-27.2%	-33.1%
95%CI	10.6–34.3	25.1–47.8	27.5–50.0	17.1–37.2	15.7–50.3
p-value	0.0003	<0.0001	0.0001	<0.0001	0.0002

**Fig 4:**
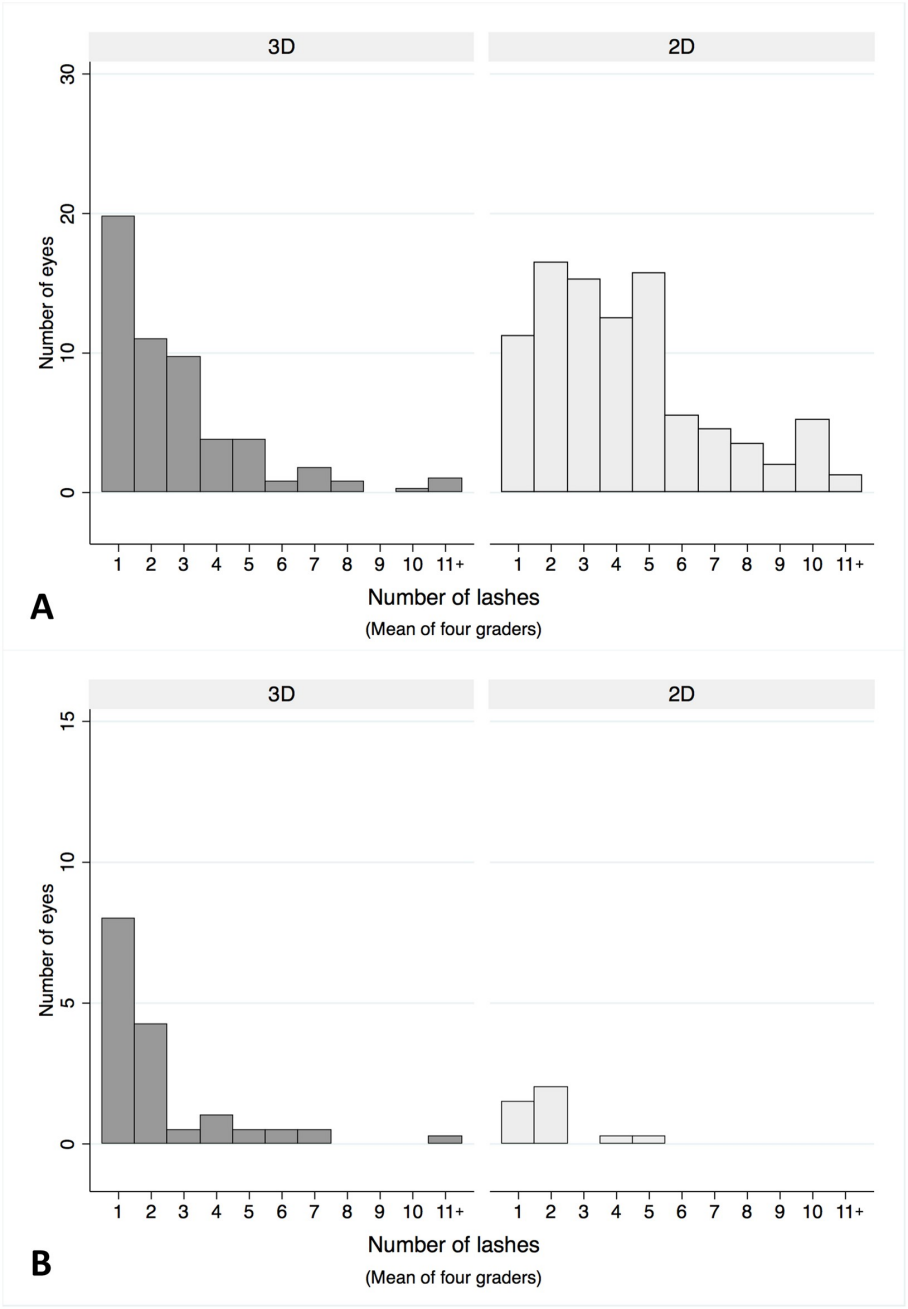
(A) False positives—distribution of the number of lashes considered to be touching from images, when no lashes were identified as touching by field grading. (B) False Negatives—distribution of the number of eyelashes touching the globe in cases of trichiasis confirmed on field grading, which were classified as not having trichiasis in the image grading (2D and 3D).

When there were discordant results between field grading and 2D or 3D photo grading, this was mainly due to false positives, i.e. lashes that were not found to be touching on field grading but were overlying the globe and therefore appeared to be touching in the images. The four graders reported fewer false positives from the 3D images. Furthermore, the false positives from 3D image grading were mainly for minor trichiasis (median 1 lash touching, IQR 0.75–2, using mean of graders), compared to 2D image grades, where the distribution was over a wider range (median 3 lashes touching, IQR 2–4.5), Fig 4A. The false negatives (cases of TT identified by field grading, but graded as not having TT by 2D or 3D images) tended to milder trichiasis, with only 1 or 2 lashes touching on field grading, Fig 4B.

### Evaluation of 3D images within a training programme

**Fig 5:**
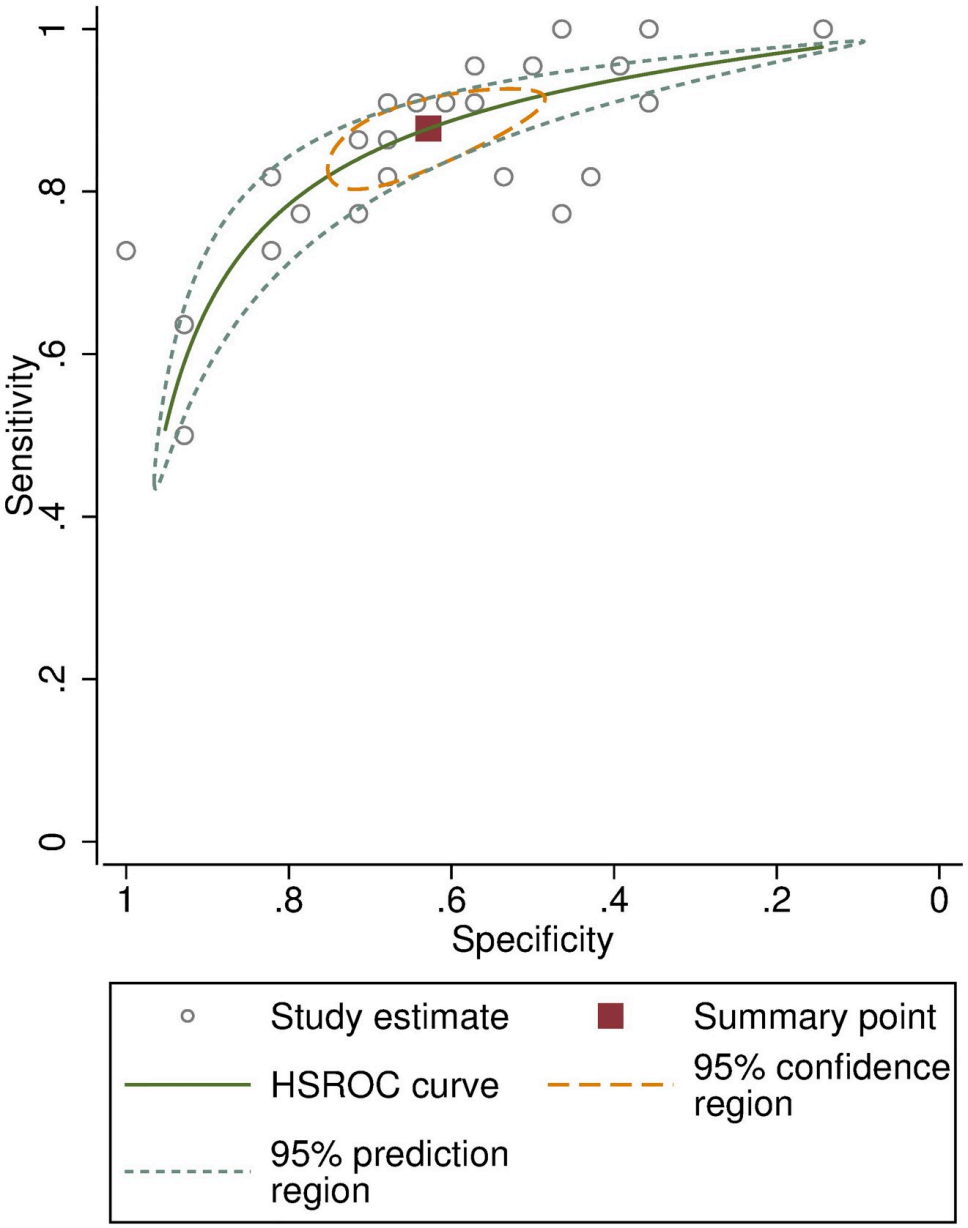
Hierarchical Summary Receiver Operating Characteristic (HSROC) curve for the trainees’ 3D image grading compared to the expert field grading.

The pooled overall agreement for the intergrader assessment comparing the trainees’ 3D image grading to the expert field grading was 71.4% (SD 9.2%, range 52–88%). The pooled sensitivity and specificity were 87.7% (CI 82.4–91.6) and 62.8% (CI 52.1–72.4), respectively, shown in the HSROC plot (Fig 5). The mean kappa score was 0.46 (CI 0.39–0.52). The individual kappa scores for each trainee are given in Table 2.

**Table 2:** Performance for each individual novice trachoma grader for the 3D image and live subject grading tests, relative to the expert grader.

ID	Training set of 3D images								Live patients							
	Sensitivity	Specificity	PPV	NPV	Agreement	Kappa	Proportion N lashes correct	Spearman correlation of lashes	Sensitivity	Specificity	PPV	NPV	Agreement	Kappa	Proportion N lashes correct	Spearman correlation of lashes
1	81.8%	82.1%	78.3%	85.2%	82.0%	0.64	64.0%	0.75	87.5%	84.6%	84.0%	88.0%	0.86	0.72	70.0%	0.80
2	81.8%	82.1%	78.3%	85.2%	82.0%	0.64	54.0%	0.67	75.0%	96.2%	94.7%	80.6%	0.86	0.72	76.0%	0.82
3	81.8%	42.9%	52.9%	75.0%	60.0%	0.23	34.0%	0.37	79.2%	92.3%	90.5%	82.8%	0.86	0.72	80.0%	0.85
4	90.9%	35.7%	52.6%	83.3%	60.0%	0.25	30.0%	0.60	83.3%	100.0%	100.0%	86.7%	0.92	0.84	74.0%	0.92
5	77.3%	46.4%	53.1%	72.2%	60.0%	0.23	32.0%	0.54	79.2%	92.3%	90.5%	82.8%	0.86	0.72	72.0%	0.88
6	95.5%	50.0%	60.0%	93.3%	70.0%	0.43	42.0%	0.52	87.5%	80.8%	80.8%	87.5%	0.84	0.68	72.0%	0.67
7	63.6%	92.9%	87.5%	76.5%	80.0%	0.58	66.0%	0.70	79.2%	96.2%	95.0%	83.3%	0.88	0.76	78.0%	0.90
8	77.3%	71.4%	68.0%	80.0%	74.0%	0.48	58.0%	0.51	100.0%	53.8%	65.7%	100.0%	0.74	0.52	70.0%	0.72
9	90.9%	60.7%	64.5%	89.5%	74.0%	0.49	46.0%	0.68	95.8%	88.5%	88.5%	95.8%	0.92	0.84	76.0%	0.77
10	77.3%	78.6%	73.9%	81.5%	78.0%	0.56	62.0%	0.66	79.2%	84.6%	82.6%	81.5%	0.82	0.64	70.0%	0.77
11	81.8%	67.9%	66.7%	82.6%	74.0%	0.48	52.0%	0.58	75.0%	88.5%	85.7%	79.3%	0.82	0.64	64.0%	0.66
12	86.4%	71.4%	70.4%	87.0%	78.0%	0.56	60.0%	0.75	87.5%	88.5%	87.5%	88.5%	0.88	0.76	76.0%	0.82
13	100.0%	14.3%	47.8%	100.0%	52.0%	0.13	20.0%	0.26	87.5%	65.4%	70.0%	85.0%	0.76	0.52	76.0%	0.81
14	81.8%	53.6%	58.1%	78.9%	66.0%	0.34	48.0%	0.49	91.7%	84.6%	84.6%	91.7%	0.88	0.76	78.0%	0.85
15	100.0%	46.4%	59.5%	100.0%	70.0%	0.43	38.0%	0.60	87.5%	88.5%	87.5%	88.5%	0.88	0.76	74.0%	0.85
16	95.5%	57.1%	63.6%	94.1%	74.0%	0.50	42.0%	0.64	79.2%	96.2%	95.0%	83.3%	0.88	0.76	74.0%	0.76
17	90.9%	67.9%	69.0%	90.5%	78.0%	0.57	62.0%	0.76	87.5%	92.3%	91.3%	88.9%	0.9	0.80	78.0%	0.88
18	86.4%	67.9%	67.9%	86.4%	76.0%	0.53	58.0%	0.61	95.8%	92.3%	92.0%	96.0%	0.94	0.88	76.0%	0.86
19	50.0%	92.9%	84.6%	70.3%	74.0%	0.45	58.0%	0.58	91.7%	92.3%	91.7%	92.3%	0.92	0.84	80.0%	0.87
20	90.9%	57.1%	62.5%	88.9%	72.0%	0.46	54.0%	0.68	95.8%	88.5%	88.5%	95.8%	0.92	0.84	80.0%	0.85
21	72.7%	82.1%	76.2%	79.3%	78.0%	0.55	56.0%	0.64	91.7%	92.3%	91.7%	92.3%	0.92	0.84	80.0%	0.88
22	100.0%	14.3%	47.8%	100.0%	52.0%	0.13	18.0%	0.41	79.2%	100.0%	100.0%	83.9%	0.9	0.80	72.0%	0.78
23	72.7%	100.0%	100.0%	82.4%	88.0%	0.75	66.0%	0.87	79.2%	76.9%	76.0%	80.0%	0.78	0.56	72.0%	0.74
24	95.5%	39.3%	55.3%	91.7%	64.0%	0.32	44.0%	0.46	95.8%	92.3%	92.0%	96.0%	0.94	0.88	78.0%	0.78
25	100.0%	35.7%	55.0%	100.0%	64.0%	0.33	28.0%	0.43	87.5%	80.8%	80.8%	87.5%	0.84	0.68	78.0%	0.87
26	90.9%	64.3%	66.7%	90.0%	76.0%	0.53	50.0%	0.70	87.5%	84.6%	84.0%	88.0%	0.86	0.72	70.0%	0.83

**Fig 6:**
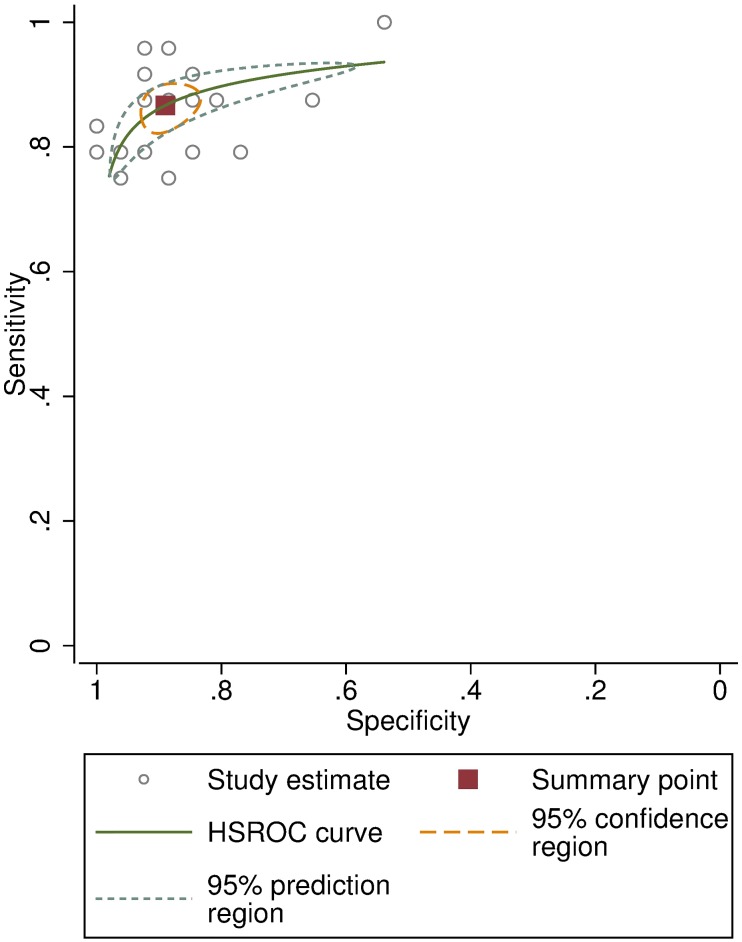
Hierarchical Summary Receiver Operating Characteristic (HSROC) curve for the trainees’ clinical grading of 50 people compared to an expert grader.

In the “live” clinical assessment test of the trainees, the pooled overall agreement was 86.8% (SD 5.3%, range 74–94%), compared to the results of an expert trachoma grader. Their pooled sensitivity was 86.7% (95%CI: 83.4–89.4) and pooled specificity was 89.0% (84.9–92.1), shown in the HSROC plot (Fig 6). The mean kappa score was 0.75 (CI 0.71–0.79). The individual kappa scores for each trainee are given in Table 2.

**Fig 7:**
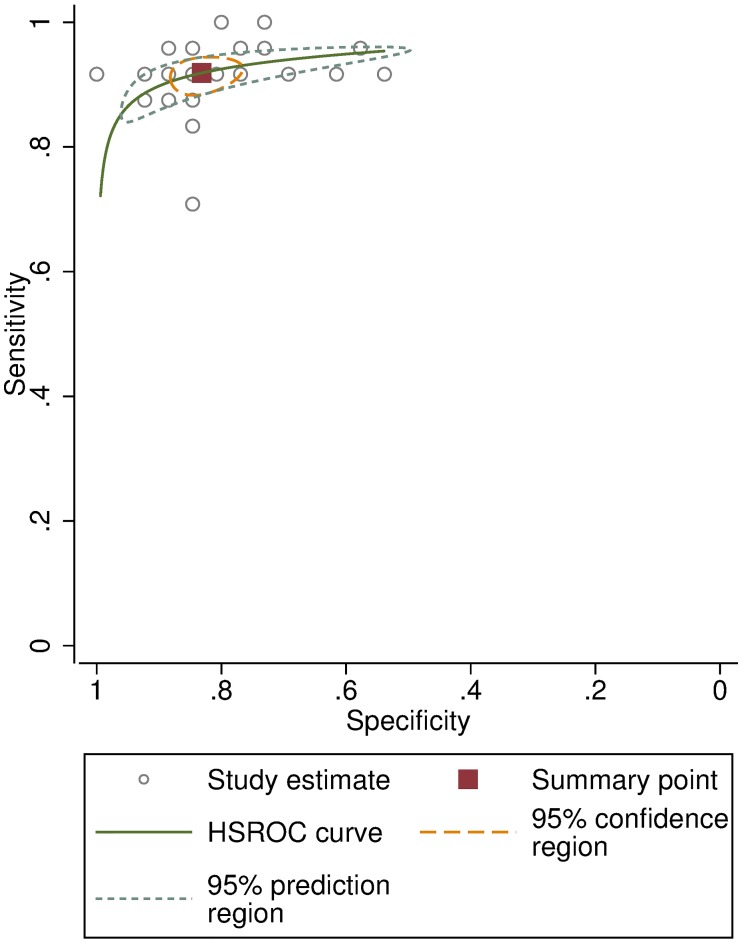
Hierarchical Summary Receiver Operating Characteristic (HSROC) curve for the experienced ICEWs’ clinical grading of 50 people compared to an expert grader.

In the “live” clinical assessment test of the experienced IECWs, the pooled overall agreement was 86.4% (SD 5.9%, range 72–96%), compared to the results of an expert trachoma grader. Their pooled sensitivity was 91.9% (95%CI: 89.3–93.9) and pooled specificity was 83.2% (78.3–87.1), shown in the HSROC plot (Fig 7). Their pooled kappa score was 0.75 (CI 0.70–0.79). The individual kappa scores for each IECW are given in Table 3.

**Table 3:** Performance for each individual experienced IECW for the live subject grading test, relative to the expert grader.

ID	Sensitivity	Specificity	PPV	NPV	Proportion Agreed	Kappa	Proportion N lashes correct	Spearman correlation of lashes
27	91.7%	100.0%	100.0%	92.9%	0.96	0.92	78.0%	0.94
28	91.7%	100.0%	100.0%	92.9%	0.96	0.92	78.0%	0.88
29	91.7%	92.3%	91.7%	92.3%	0.92	0.84	76.0%	0.78
30	100.0%	73.1%	77.4%	100.0%	0.86	0.72	66.0%	0.82
31	91.7%	92.3%	91.7%	92.3%	0.92	0.84	80.0%	0.91
32	91.7%	53.8%	64.7%	87.5%	0.72	0.45	52.0%	0.74
33	91.7%	76.9%	78.6%	90.9%	0.84	0.68	66.0%	0.76
34	91.7%	69.2%	73.3%	90.0%	0.8	0.60	64.0%	0.74
35	91.7%	61.5%	68.8%	88.9%	0.76	0.53	44.0%	0.52
36	91.7%	80.8%	81.5%	91.3%	0.86	0.72	70.0%	0.81
37	91.7%	80.8%	81.5%	91.3%	0.86	0.72	60.0%	0.69
38	87.5%	84.6%	84.0%	88.0%	0.86	0.72	70.0%	0.74
39	95.8%	88.5%	88.5%	95.8%	0.92	0.84	84.0%	0.95
40	70.8%	84.6%	81.0%	75.9%	0.78	0.56	62.0%	0.60
41	95.8%	57.7%	67.6%	93.8%	0.76	0.53	46.0%	0.47
42	95.8%	76.9%	79.3%	95.2%	0.86	0.72	66.0%	0.73
43	91.7%	84.6%	84.6%	91.7%	0.88	0.76	64.0%	0.72
44	100.0%	73.1%	77.4%	100.0%	0.86	0.72	70.0%	0.81
45	83.3%	84.6%	83.3%	84.6%	0.84	0.68	60.0%	0.66
46	87.5%	88.5%	87.5%	88.5%	0.88	0.76	72.0%	0.84
47	91.7%	92.3%	91.7%	92.3%	0.92	0.84	74.0%	0.79
48	95.8%	84.6%	85.2%	95.7%	0.9	0.80	68.0%	0.80
49	91.7%	88.5%	88.0%	92.0%	0.9	0.80	70.0%	0.86
50	87.5%	92.3%	91.3%	88.9%	0.9	0.80	74.0%	0.79
51	87.5%	88.5%	87.5%	88.5%	0.88	0.76	74.0%	0.84
52	100.0%	80.0%	82.8%	100.0%	0.88	0.80	56.0%	0.66
53	95.8%	73.1%	76.7%	95.0%	0.84	0.68	64.0%	0.65

There was no evidence of a difference between the trainees and the experienced IECWs in the odds of overall correctly diagnosing the presence or absence of TT (OR = 0.96, 95%CI 0.74–1.24, p = 0.76). However, there was some evidence that the two groups had slightly different sensitivity and specificity. The experienced IECWs had slightly higher sensitivity (OR = 1.74, 95%CI 1.21–2.49, p = 0.003) for detecting TT than the trainees. Conversely, the IECWs had a slightly lower specificity (OR = 0.62, 95%CI 0.39–0.98, p = 0.041) than the trainees.

The number of trichiasis cases and the number of eyelashes touching the cornea did not change between the first grading by the trachoma expert and the final grading at the end of testing.

The 3D glasses were found to be either “very easy” or “easy” to use by 80.8% of trainees and viewing the 3D images was considered to be either “very realistic” or “realistic” by 84.6% of the trainees (Table 4). All 26 (100%) trainee participants found the 3D images were more useful than 2D images for training and thought that they should be included in future training. The most commonly reported reasons for 3D image preference included being able to see trichiasis more clearly than with 2D images (52% of responses) and being easy to use (26% of responses). Conversely, negative feedback for 3D images centred around taking time to get used to viewing them (62.1%), with the second most frequently reported negative feature was being difficult or unable to use (17.2%). When asked for suggestions for improvement with the 3D training, the most commonly suggested improvement was to allow for more time for 3D training (22.6%), whilst no improvement was suggested in 41.9% of responses. The full results are given in Table 4.

**Table 4:** Results from feedback from trachoma grader participants (% of responses).

Question / Response Options	%
**How did you find using 3D glasses to use?**	
Very difficult / unable	0
Difficult	7.7
Neither difficult or easy	11.5
Easy	69.3
Very Easy	11.5
**Did you find using 3D photography realistic?**	
Very unrealistic	0
Unrealistic	0
No difference	15.4
Realistic	65.4
Very realistic	19.2
**Which do you find more useful as a training tool?**	
2D	0
3D	100
**Should 3D images be used in future training programmes?**	
Yes	100
No	0
**What did you like most about using 3D images for training?**	
Realistic	12
Easy to use	26
Able to see TT better than 2D	52
Useful tool for TT training	10
**What did you dislike most about using 3D images for training?**	
Nothing	10.3
Unable / difficult to use	17.2
2D photos are clearer	3.4
Took time to get used to it	62.1
Not realistic	3.4
Difficult to identify epilated lashes	3.4
**Suggestions for improvement**	
Computers for training and exam rather than printed images	9.7
3D viewing glasses with more magnification	6.5
Distribute 3D glasses to healthcare facilities	3.2
More time for training with 3D photos	22.6
Study impact of viewing through 3D glasses on eyes for a long period of time	6.5
Clearer and larger photos	3.2
Use natural light while grading	3.2
Make using 3D glasses easier	3.2
Nothing to improve / no suggestion	41.9

## Discussion

It is currently estimated that there are about 3.2 million people with TT in need of surgery.[17] Reliably identifying people with TT is key for both finding all individuals needing corrective trichiasis surgery, as well as measuring programme impact and ultimately the validation of elimination of trachoma as a public health problem by WHO. In many settings the numbers of individuals with untreated TT are already very low or anticipated to become very low in the near future. Therefore, it is increasingly impractical to gather sufficient numbers of TT cases together at one time to perform a “live” interobserver assessment exercise. Reliable alternative methods to train and assess TT graders are needed.

In this study we first investigated the relative utility of 2D and 3D images of the same eyes to determine whether or not eyelashes were touching the eye. Using field grading as the “gold standard”, the sensitivity of 3D photography was high (88%) and its specificity was relatively good (60%). Although the sensitivity of 2D image grading was higher (98%) than for 3D, this was at a considerable cost to specificity (27%). There was a tendency for the graders to “overcall” TT from 2D images; this was significantly reduced by using 3D images. In 2D images downward projecting eyelashes from the upper eyelid may appear to be touching the eye when in fact they are not. We found that 3D images help to overcome this to a certain extent by providing a stereoscopic view of the eyelashes, so that it is easier to tell if they are touching the eye or projecting over it without touching. The kappa scores and percent agreement were higher for 3D images, providing some evidence that these are moderately better than 2D images for determining whether or not lashes are touching the eye.

There is little published data comparing photographic grading with field grading for TT. Most studies have focused on grading active trachoma.[11–14] Moreover, there is little published on the reliability of TT grading from an operational setting. We recently reported use of 2D eyelid images to assess for signs of TT; this was done to independently evaluate potential observer bias when assessing the outcome of two different surgical interventions in a randomised controlled trial.[15] Although there was good agreement between the field and image grading (% agreement, 86.6%; Kappa, 0.60; Sensitivity, 83.8%; Specificity, 87.2%, PPV, 58%; NPV, 96.2%), we found 2D image grades tended to slightly “overcall” TT, which is consistent with the present study, and led us to explore the potential value of 3D photography for this. There have been no previous studies assessing 3D photography for TT.

An analysis of the number of lashes thought to be touching in TT “false positive” eyes from 3D images found the large majority had only 1 or 2 lashes, when the field grading indicated none. The “false positive” eyes from the 2D images had a higher median value. Again, this is likely due to the greater difficulty of assessing the relative position of lashes in a two-dimensional image. The “false negatives” tended to be eyes with only 1 or 2 lashes touching.

Three of the four graders had similar results. However, Grader 3 tended to “overcall” trichiasis for both image sets, leading to a lower specificity. This accounts for the wide confidence interval for pooled specificity. Despite this, the specificity and percent agreement for Grader 3 were higher for the 3D images, suggesting that this provides more reliable differentiation than 2D images.

Training with 3D images was well received by the candidates. They gave positive feedback, and it prepared them well for grading patients. The performance of the new trainees who had been taught on 3D images and the experienced IECWs were very comparable.

The trainees performed better in the “live” grading exercise than on the 3D assessment. This suggests that the 3D test may in fact be more difficult than the field grading itself. Furthermore, candidates received their results with feedback following the 3D test, which may have influenced how they performed on the field grading test (i.e. candidates performing poorly may have been motivated to prepare better for the field grading or improved their skills as a result of the exercise). The results of the 3D test do not predict performance in the field grading.

Tropical Data uses a cut-off of 0.7 or greater as the kappa score for the IGA test for TF, for both the slide test as well as on real patients. [9,10] There is currently no formal assessment of TT grading in the prevalence survey training manual, although the OSCE methodology used in the TT-only training manual will be incorporated into the upcoming revised prevalence survey training manual. If we were to use the same benchmark, only 1/26 candidates would “pass” the 3D IGA test (and none of the four experts). We therefore do not recommend that the results of the 3D IGA test should be used for TG to progress to field grading. However, the TG performed much better with the real patients, when 19/26 TG would have passed this benchmark. We therefore propose that training candidates using 3D photography is a useful, more realistic tool than 2D photography alone.

There are a number of limitations to our study. In this study the field grading was considered to be the “gold standard”, which assumes that all cases of trichiasis and no trichiasis were correctly diagnosed. It is possible, although relatively unlikely, that the field grading may have been incorrect in some cases, which would affect the results. This field grader (EH) has more than 10 years field grading experience and has previously been shown to have a very strong agreement in grading validation studies with senior graders.[15]

For the 2D photograph in this study, we chose to use the left-hand image of the split 3D image, rather than the higher resolution 2D macro image which was available. It was felt that although the macro image was of a higher resolution and much higher magnification, it was less realistic compared to what would be encountered in real life and therefore of less value for training and assessment purposes. This study set out to assess a new form of imaging for training and assessing graders rather than for remote image grading. Using 3D viewing glasses can take a few minutes of practice and requires binocular single vision. The 2D image used was not taken using the same set-up as previous studies using a dedicated 2D macro lens which gives much greater magnification. The results for 2D grading may be better when using a more magnified image; however, as discussed above, this is less realistic when compared to examining in the community. There is a limitation to using Kappa scores as its use depends partly on the proportion of the population on which it is difficult to agree, with lower Kappa scores when there is a higher number of difficult cases.[18]

We did not directly compare novice TG trained using conventional 3D images to a separate group of novice TG trained using 2D images as we had previously shown that 3D images provide similar sensitivity and substantially better specificity for the detection of trichiasis.

Stereopsis is required to view images in 3D and to examine patients in 3D. It is estimated that 5% have no stereopsis and 32% have moderate to poor stereopsis.[19] We did not formally assess the participants’ stereopsis as this is not usually done as part of the selection process of trachoma graders. It is possible that some trainees might have been unable to for a 3D view, however, that would have also likely been the case for their live examinations. It may be appropriate for programmes to evaluate stereopsis before training.

In the real-life grading the examiner is able to move around the patient to assess for any lashes touching the eye from different angles. In particular, asking the patient to look up and looking from the side can be helpful. In an attempt to emulate this, we did pilot taking photographs from the side. However, the limitation we found was the depth of field was such that only a small length of the upper lid lashes was in focus at the same time really limiting its value.

In conclusion, we think that using standardised 3D images of TT can be a useful tool in training trachoma graders to identify TT, with a specificity performance that is better than that of 2D image grading, and leads to live examination results that are comparable to those of experienced graders.
